# Draft genome sequence of *Tetramitus* sp. JP001

**DOI:** 10.1128/mra.00797-25

**Published:** 2025-10-03

**Authors:** Atham Hari Naga Papa Rao, Lakshmanan Vighnesh, Himanshi Bhagat, Chintalapati Sasikala, Chintalapati Venkata Ramana

**Affiliations:** 1 Department of Plant Sciences, School of Life Sciences, University of Hyderabad, P.O. Central University 98773, Hyderabad, India; 2 Smart Microbiology Services, Hyderabad, Telangana, India; Rochester Institute of Technology, Rochester, New York, USA

**Keywords:** protist, *Tetramitus*, MAG, draft genome, bacterial pathogens

## Abstract

We report a 34.6 Mb draft genome of *Tetramitus* sp. JP001, which was isolated from the rhizoplane of a freshwater *Marsilea* sp. using *Escherichia coli* JC875 as prey. Despite their biological and ecological implications, members of the *Tetramitus* genus are not as extensively studied as other protists.

## ANNOUNCEMENT

**Fig 1:**
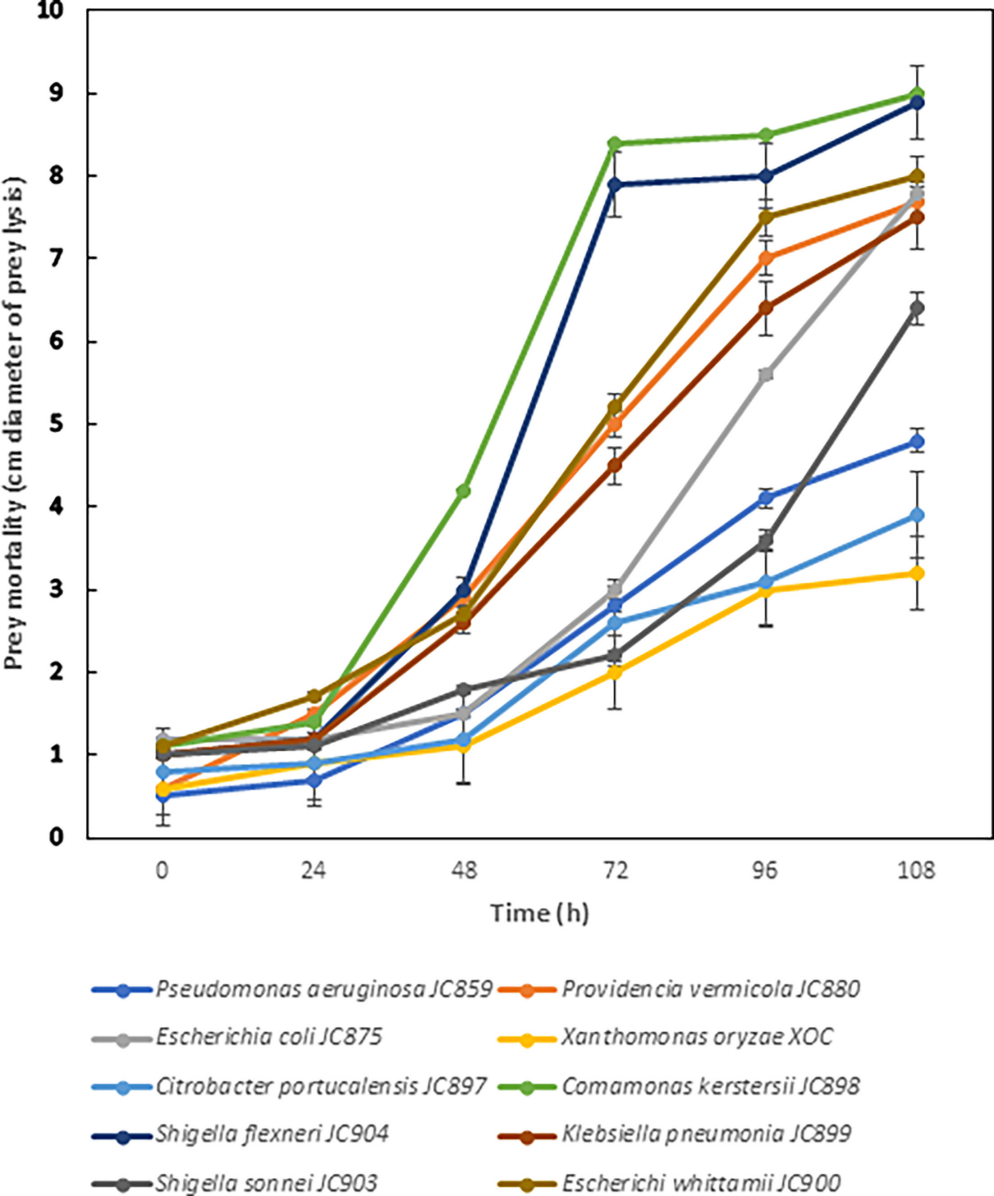
Predation kinetics of strain JP001 on different bacterial prey as measured by the zone of prey lysis.

*Tetramitus* is a protozoan that belongs to the *Vahlkampfiidae* family and can predate on bacteria, yeast, and other protozoans. These members are inhabitants of freshwater and soil (1) and help in the maintenance of ecological balance and promote plant growth (2). This study reports the draft genome of a *Tetramitus* sp. strain JP001, which predates on some of the plant and animal pathogens (Fig. 1) and can be a candidate live antibiotic (3).

*Tetramitus* sp. JP001 was isolated from a freshwater *Marsilea* sp., from the University of Hyderabad, Hyderabad, India (17.4578°N 78.3182°E). *Escherichia coli* JC875 lawn was prepared on L.B (Luria broth, HIMEDIA, M575) plate by incubating at 37°C for 24 h. On the surface of the lawn, the *Marsilea* sp. root sample (1 cm×1 cm) was inoculated. Lysis of the prey was observed after 7 days of incubation as a clear zone and increased with time. For Shotgun sequencing, the sample was prepared by culturing *Tetramitus* sp. on *E. coli* lawn at 37°C for 96 h. The lysed zone of the prey was scraped and suspended in 1 mL of saline and was outsourced to Allele Tech Private Limited, Hyderabad, for DNA extraction and sequencing. The DNA was extracted using the QIAGEN Bacterial Extraction Kit as per the manufacturer’s instructions. The Nano Photometer N60 and Qubit DNA HS Assay Kit were used for DNA quantification. The library was prepared using the QIAseq FX DNA Library Core Kit, and the paired-end (2×150 bp) sequencing was carried out by Illumina’s NovoSeq 6000 platform. A total of 25,054,035 reads were obtained, which were then processed using fastp v0.23.4 (4). *E. coli* reads were removed by using BBduk (5) by giving *E. coli* DSM 30083 as the reference genome. The decontaminated reads from BBduk were used for assembly generation. MEGAHIT v1.2.9 (6) was used to assemble the reads. The resulting assembly was then binned using MaxBin2 v2.2.4 (7), which generated a metagenome-assembled genome (MAG). All tools and software used for analyzes were either performed on the Galaxy server (8) or the Kbase platform (9) with the default parameters. Barrnap 0.9 (10) was used to retrieve rRNA gene sequences from the MAG. The 18S rRNA gene sequence (2139 bp) was retrieved from the genome, which showed the highest identity of 98.3% with *Tetramitus lobospinosus* ATCC30298; however, the genome sequence of this strain is not available.

The genome size of strain JP001 is 34.6 Mb, and the GC content is 39%. There are 8,354 contigs with an N50 value of 4.5 kb. The assembly was mapped back to the decontaminated reads using Bowtie2 (11), which showed a 63.73% overall alignment and 67.77 mean coverage. BUSCO v5.8.0 (12) analysis was carried out for the genome. Out of the 255 BUSCOs in the eukaryota_odb10 lineage data set, 170 complete BUSCOs were present in the genome of strain JP001.

## Data Availability

The MAG has been deposited in GenBank under the accession number JBNIDJ000000000, along with BioProject PRJNA1192423, BioSample SAMN45138060, and SRA SRX26944924.
